# Representations of naturalistic stimulus complexity in early and associative visual and auditory cortices

**DOI:** 10.1038/s41598-018-21636-y

**Published:** 2018-02-21

**Authors:** Yağmur Güçlütürk, Umut Güçlü, Marcel van Gerven, Rob van Lier

**Affiliations:** Donders Institute for Brain, Cognition and Behaviour, Radboud University, Nijmegen, The Netherlands

## Abstract

The complexity of sensory stimuli has an important role in perception and cognition. However, its neural representation is not well understood. Here, we characterize the representations of naturalistic visual and auditory stimulus complexity in early and associative visual and auditory cortices. This is realized by means of encoding and decoding analyses of two fMRI datasets in the visual and auditory modalities. Our results implicate most early and some associative sensory areas in representing the complexity of naturalistic sensory stimuli. For example, parahippocampal place area, which was previously shown to represent scene features, is shown to also represent scene complexity. Similarly, posterior regions of superior temporal gyrus and superior temporal sulcus, which were previously shown to represent syntactic (language) complexity, are shown to also represent music (auditory) complexity. Furthermore, our results suggest the existence of gradients in sensitivity to naturalistic sensory stimulus complexity in these areas.

## Introduction

Early research in perception identified stimulus complexity as one of the most important stimulus properties that affect task performance^1^. In the visual domain, stimulus complexity has been shown to influence time perception^2^, speed and accuracy of shape recognition^3^, amodal completion^4,5^ the reaction time of search, discrimination and recognition tasks^6–10^, as well as affecting memory^11,12^ and perceptual learning^13,14^ performance. Furthermore, complexity relates to interestingness, pleasantness, liking and similar subjective aesthetic evaluations of art^15^, natural images of scenes^16^ and architecture^17^. It has been much studied in the domain of art perception and empirical aesthetics^18–22^ as well as environmental psychology^16,23^ for its role in preferences. Recently, its effect on neural decoding of visual stimuli from brain activity has also been investigated^24^. Similarly in the auditory domain, complexity plays an important role in stimulus perception with its modulatory effects on attention, arousal and memory^25^. Furthermore, as is the case with its visual counterpart, music complexity highly influences whether a song will be liked or not^19,26–29^.

Borrowing ideas from information theory^30^, complexity of a stimulus is commonly thought as the amount of information contained in it^31^. However, moving away from information theory, when the perceptual limitations of humans are embedded into its definition, complexity becomes a subjective and multi-faceted concept^1,18,32^. In the case of visual complexity, the dimensions making up the complexity of a stimulus include properties such as regularity, number of elements, and diversity^15^. Similarly, according to structural information theory, complexity (or simplicity) is defined in terms of the regularity of patterns, which depends on iteration, symmetry and alternation properties^33–35^. However, for natural images such as photographs of scenes, objects or works of art, it is difficult to correctly determine such properties. In these cases, computational measures of complexity become useful and can be evaluated automatically instead of using the aforementioned elements to define an intractable complexity measure. Specifically, measures related to information content such as Kolmogorov complexity estimated as compressed file size of images^9,19,36,37^, self-similarity and fractal dimension^38–41^, and Pyramid of Histograms of Orientation Gradients (PHOG) derived measures^20,42^ have been frequently used to automatically obtain an estimate of subjective complexity of images. In the context of music, complexity is a highly subjective term that encompasses several properties^31^, such as tempo, predictability, variety of instruments, harmony and rhythm^43^. However, recent models of music complexity suggest that the best predictors of subjective complexity of a song are the event density and Kolmogorov complexity estimated as the compressed file size of the song^19^.

Despite its importance, functional neuroimaging studies investigating the neural correlates of stimulus complexity have been limited, especially for naturalistic stimuli. In the visual domain, electrophysiological investigations showed that the amplitude of the N2 event-related potential and late positive potential increased with increased complexity of randomly generated polygons^44^. One of the first functional brain imaging studies that measured cerebral metabolic rate for glucose using positron emission tomography (PET) determined that as stimulus complexity increased (from white light to an alternating checkerboard pattern) the glucose metabolic rate also increased in both the primary and associative visual cortices, but faster in the associative visual cortex^45^. Similarly, using PET, it was shown that cerebral blood flow velocity in the occipital cortex increased as visual stimuli from different categories with increasing complexity (diffuse light, checkerboard pattern and movie) is presented to subjects^46^. In the case of auditory stimulus complexity, a meta-analysis investigated where the different categories of sounds such as pure tones, noise, music and vocal sounds caused activations across the human brain^47^. In a different study, it was shown that brain connectivity patterns of people listening to their favorite songs were consistent with each other, despite the differences between their favorite songs in terms of complexity^48^.

The current study aims to provide a comprehensive account of representations of naturalistic sensory stimulus complexity in sensory cortices by establishing a direct, predictive relationship between objective stimulus complexity measures and stimulus-evoked brain activations as well as characterizing the properties of this relationship under the framework of neural encoding and decoding in functional magnetic resonance imaging (fMRI)^49^. That is, we develop several neural encoding and decoding models, which embody specific hypotheses about certain stimulus features modulating stimulus-evoked brain activations, to test alternative hypotheses about what, if any, stimulus complexity measures are represented in different brain regions as well as analyzing these models to characterize the properties of these representations. To this end, we present the results of two different fMRI experiments; one with naturalistic image stimuli to characterize the representations of visual complexity and another with music stimuli to determine the those of auditory/music complexity in the brain. Our results reveal four core findings regarding the processing of stimulus complexity in the sensory cortices: i) Stimulus complexity was shown to modulate visual and auditory cortices. ii) A quantification of the complexity sensitivity of individual regions of interest (ROIs) demonstrated a change of sensitivity (from fine grained to coarser) in a gradient from lower to higher areas. iii) It was shown that parahippocampal place area (PPA) has distributed representations of complexity comparable to or better than the ROIs in the early visual cortex, supporting the notion that global scene properties such as complexity plays an important role in scene processing. iv) It was shown that regions of the auditory cortex, which represent syntactic language complexity such as posterior regions of superior temporal gyrus (STG) and superior temporal sulcus (STS), also represent music complexity.

## Methods

### Ethics Statements

Experiment 1 was approved by the Ethics Committee of ATR and the subjects provided written informed consent. Experiment 2 was approved by the local ethics committee (CMO Regio Arnhem-Nijmegen) and the subjects provided written informed consent in accordance with the Declaration of Helsinki. All experiments were performed in accordance with the relevant guidelines and regulations.

### Experiment 1

#### Dataset

In the first experiment, we used a preexisting dataset^50^. Here, we report only the most pertinent details. Additional details can be found in the original publication. The dataset comprises visual stimuli (photographs) and fMRI data of five healthy adult subjects (23–38 year old one female and four male subjects with normal or corrected-to-normal vision).

Design. The dataset comes in two parts [The dataset contains also an imagery set, which is not considered here.]:

i) A training set that was collected in 25 unique runs over multiple sessions, which consist of 50 unique and 5 repeated trials per run. In one training run, 51 different images were presented. Fifty of these images were repeated only once within the run. One of these images were repeated five times within the run. Each of the 25 training runs used a different set of images.
ii) A test set that was collected in 35 repeated runs over multiple sessions, which consist of 50 unique and 5 repeated trials per run. Like the training set, in one test run, 51 different images were presented. Fifty of these images were repeated only once within the run. One of these images were repeated five times within the run. Unlike the training set, each of the 35 test runs used the same set of images.

The repeated images were used to facilitate a one-back task and were not included in the final data set. After the exclusion of the repeated images, the training set ended up with 1250 different images repeated once each, while the test set ended up with 50 different images repeated 35 times each.

Each image was presented at 12° × 12° and 5 Hz for 9 s, which was followed by the one-back repetition detection task. Subjects fixated a central point throughout each run.

Visual stimuli. The stimuli were drawn from the subset of the original ImageNet dataset^51^ that was used in the Large Scale Visual Recognition Challenge 2011^52^ (ILSVRC2011). The subset comprises 1350000 photographs, each of which belongs to one out of 1000 categories. The training set contains 1200 stimuli that belong to 150 representative categories (eight photographs per category). The test set contains 50 stimuli that belong to 50 representative categories (one photograph per category). The categories are mutually exclusive. A list of these 200 categories are available as Supplementary Information. Example categories include bathtub, chimpanzee, fire truck, human being, hot-air balloon, watermelon, etc. Each stimulus was center cropped to the largest square possible and resized to 500 px × 500 px with antialiasing and bicubic interpolation.

MRI data. MRI data were acquired with a Siemens 3 T MAGNETOM Trio scanner. Anatomical scans were collected with T1-weighted MP RAGE and T2-weighted turbo spin echo pulse sequences. Functional scans were collected with a T2*-weighted gradient echo echo planar imaging pulse sequence (voxel size: 3 mm^3^; slices: 50 for localizer and task scans, and 30 for retinotopy scans; distance factor: 0%; FoV read: 192 mm; TR: 3000 ms for localizer and task scans, and 2000 ms for retinotopy scans; TE: 30 ms; flip angle: 80 degrees; multi-slice mode: interleaved).

The fMRI data were preprocessed as follows: The functional scans were realigned to one another and coregistered to the anatomical scans. The realigned and coregistered functional scans in each run were linearly detrended and standardized along the time axis. The realigned functional scans in each trial were shifted by 3 s (one TR), cropped to the first 9 s (3 TRs) and averaged along the time axis. This resulted in one time point per trial per voxel.

Seven ROIs in the lower visual cortex and the higher visual cortex were defined based on retinotopic mapping and functional localizers. Table 1 shows the details of these ROIs.

**Table 1:** ROIs considered in the first (visual) experiment.

Area	Also known as	Region	References
V1 (Primary Visual Cortex)	17, hOC1, OC, BA17	Lower Visual Cortex	^78,79^
V2 (Second Visual Area)	18, hOC2, OB, BA18	Lower Visual Cortex	^78,79^
V3 (Third Visual Area)	V3d, V3v, VP, hOC3d, hOC3v	Lower Visual Cortex	^78,79^
V4 (Fourth Visual Area)	V4d, V4v, hV4, hOC4v, hOC4lp, LO1	—	^78,79^
LOC (Lateral Occipital Complex)	LO1, LO2, hOC4la	Higher Visual Cortex	^80^
FFA (Fusiform Face Area)	FFC, FG2	Higher Visual Cortex	^81^
PPA (Parahippocampal Place Area)	—	Higher Visual Cortex	^67^

#### Visual complexity measures

The complexity of the visual stimuli was parameterized with three different measures. These computational visual complexity measures are objective measures of complexity, which are estimates of subjective complexity. These exact measures have been shown to reflect the subjective complexity levels of images in earlier behavioral studies^20,22,42^.

- **Mean maximum magnitude gradient** (**Gradient**)**:** This measure was based on the ‘Complexity’ measure as described in a previous study^42^. It measures the maximum rate of change in the Lab color space channels of an image. It was computed as follows^42^:
  1 $$\mathop{{\rm{mean}}}\limits_{x,y}\,\mathop{{\rm{\max }}}\limits_{c}\parallel \nabla {S}_{c}(x,y)\parallel $$
  where *S*_*c*_(*x*, *y*) is the pixel (*x*, *y*) in the channel *c* of the stimulus in the Lab color space such that *c* ∈ {*L*, *a*, *b*}, *x* ∈ {1, …, 256} and *y* ∈ {1, …, 256}.
- **Portable Network Graphics** (**PNG**)**:** The PNG-based complexity measure of a stimulus was computed as the compressed (lossless) file size (bytes) of the stimulus in the RGB color space. This measure can be thought of as a surrogate for the Kolmogorov complexity of the image, which is defined as the length of the shortest computer program or algorithm that can be used to represent the object^53^. It essentially gives an indication of the information content of the image. PNG encoding was carried out using *Pillow 4*.*1*.*1* (https://python-pillow.org/) with default parameter settings.
- **Self**-**similarity:** Self-similarity is a measure of how much the whole of an object resembles its parts. To estimate this for each stimulus image, we compared histograms of oriented gradients (HOGs) of the whole image at the ground level and subregions of the image at the third level^42^. Specifically, the self similarity-based complexity measure of a stimulus was computed as follows^42^:

2 $$\mathop{{\rm{median}}}\limits_{f}\,(\sum _{x=1}^{8}\,\sum _{y=1}^{8}\,{\rm{\min }}\,({{\rm{PHOG}}}_{\lceil x\mathrm{/2}\rceil ,\lceil y\mathrm{/2}\rceil }^{\mathrm{(2)}}(f),{{\rm{PHOG}}}_{x,y}^{\mathrm{(3)}}(f)))$$

where ${{\rm{PHOG}}}_{x,y}^{(l)}(f)$ is the feature *f* in the subregion (*x*, *y*) and the level *l* of the pyramid histogram of oriented gradients^54,55^ of the stimulus in the Lab color space with *f* ∈ {1, …, 16} (16 equally spaced orientations between −*π* radians and *π* radians).

Note that all visual stimuli were resized to 256 px × 256 px with Lanczos interpolation and converted to the RGB color space (if required) prior to the computation of the visual complexity measures.

### Experiment 2

For the second experiment, we used a new dataset, which comprises auditory stimuli (music) and fMRI data of eight healthy adult subjects (24–38 year old four female and four male subjects with normal hearing).

#### Design

Subjects participated in two sessions: one for a training set and another one for a test set. The training set was collected in eight unique runs, each of which consisted of 16 unique trials. The test set was collected in eight repeated runs, each of which consisted of 16 unique trials.

In each trial, a stimulus was presented for 29 s with in-ear headphones, followed by a self-paced complexity preference rating task. Subjects fixated a central point throughout each trial. The task was used to keep the subjects engaged, and the ratings were excluded from the analyses.

Prior to entering the scanner, subjects listened to three example stimuli: a very loud one, a normal one and a very quite one. After subjects entered the scanner and before the first run, the second example stimulus was presented for 29 s with in-ear headphones (while fMRI data were acquired, which were later discarded). During this period, subjects adjusted the volume to a comfortable level.

#### Auditory stimuli

We used 144 auditory stimuli that were systematically drawn from the MagTag5k Autotagging Dataset^56^ – a preprocessed version of the original MagnaTagATune Dataset^57^, which solves some of the problems in the original such as duplication and synonymy. The preprocessed dataset contains 5259 29-second long, 16000 Hz song excerpts and 136 binary tags per excerpt.

In order to create a stimulus dataset that spanned a large musical spectrum based on their associated tags, We used hierarchical (agglomerative) clustering with correlation distance (1 - Pearson correlation coefficient) and complete linkage to cluster all song excerpts to 16 clusters based on the binary tags. Among all excerpts in each cluster, the one with the highest within-cluster similarity was assigned to the test set. This resulted in a test set of 16 stimuli (=16 clusters × 1 excerpt). Among remaining excerpts in each cluster, eight with the highest within-cluster similarity were assigned to the training set. This resulted in a training set of 128 stimuli (=16 clusters × 8 excerpts). A list of all 59 tags that were associated to the 144 stimuli are available as Supplementary Information. Example tags include ambient, electro, instrumental, female vocal, piano, strange, etc.

#### MRI data

MRI data were acquired with Siemens 3 T MAGNETOM Prismafit scanner and Siemens 32-Channel Head Coil. Anatomical scans were collected with a T1-weighted MP RAGE pulse sequence (voxel size: 1 mm^3^; slabs: 1; distance factor: 50%; orientation: sagittal; FoV read: 256 mm; slice thickness: 1 mm; TR: 2300 ms; TE: 3.03 ms; PAT mode: GRAPPA; accel. factor PE: 2; flip angle: 8 degrees; multi-slice mode: single shot). Functional scans were collected with a T2*-weighted gradient echo echo planar imaging pulse sequence (voxel size: 2 mm^3^; slices: 64; distance factor: 0%; orientation: transversal; FoV read: 210 mm; slice thickness: 2.4 mm; TR: 735 ms; TE: 39 ms; multi-band accel. factor: 8; flip angle: 75 degrees; multi-slice mode: interleaved).

The fMRI data were preprocessed as follows: The functional scans were realigned to the first functional scan and the mean functional scan, respectively. Realigned functional scans were slice time corrected. The realigned and slice time corrected functional scans in each run were linearly detrended and standardized along the time axis. The realigned and slice time corrected functional scans in each trial were shifted by 2.94 s (four TRs), cropped to 29.4 s (40 TRs; approximately one stimulus) and averaged with a window size of 8.82 s (12 TRs) and a hop size of 0.735 s (one TR) along the time axis. This resulted in 28 time points per trial.

Thirteen ROIs in the early auditory cortex and the auditory association cortex were defined based on the HCP MMP 1.0 parcellation^58^ after projecting it to the native volumetric space via HCP MMP 1.0 parcellation → fsaverage surface space → native surface space → native (anatomical) volumetric space → native (functional) volumetric space. Table 2 shows the details of these ROIs.

**Table 2:** ROIs considered in the second (auditory) experiment.

Area	Also known as	Region	References
A1 (Primary Auditory Cortex)	Core, R1, TC, TE1.0, TE1.1, 41	Early Auditory Cortex	^58,82–85^
LBelt (Lateral Belt Complex)	Belt, TB	Early Auditory Cortex	^58,83–85^
MBelt (Medial Belt Complex)	Belt, TB	Early Auditory Cortex	^58,83–85^
PBelt (ParaBelt Complex)	ParaBelt, TA1	Early Auditory Cortex	^58,83–85^
RI (RetroInsular Cortex)	reI, reIt, RetroInsular, Belt, TD	Early Auditory Cortex	^58,82,84–87^
A4 (Auditory 4 Complex)	TE3	Auditory Association Cortex	^58,88^
A5 (Auditory 5 Complex)		Auditory Association Cortex	^58^
STSdp (Area STSd posterior)		Auditory Association Cortex	^58^
STSda (Area STSd anterior)		Auditory Association Cortex	^58^
STSvp (Area STSv posterior)		Auditory Association Cortex	^58^
STSva (Area STSv anterior)		Auditory Association Cortex	^58^
STGa (Area STGa)		Auditory Association Cortex	^58^
TA2 (Area TA2)	TE1.2	Auditory Association Cortex	^58,84,85,89^

#### Auditory complexity measures

The complexity of the auditory stimuli was parameterized with three different measures. These computational auditory complexity measures are objective measures of complexity, which are estimates of subjective complexity. These exact measures have been shown to reflect the subjective complexity levels of songs in an earlier comprehensive behavioral study analyzing the relationship between various subjective and objective stimulus complexity measures^19^.

- **Free Lossless Audio Codec file size** (**FLAC**)**:** The compressed file size (in bytes) of the stimuli with a lossless audio codec. This measure can be thought of as the Kolmogorov complexity of the stimuli, similar to the PNG measure in the case of the visual stimuli. FLAC encoding was carried out using *ffmpeg* (https://ffmpeg.org/) with 16000 samples per second and 16 bits per sample.
- **Ogg Vorbis file size** (**Ogg**)**:** The compressed file size (in bytes) of the stimuli with a lossy audio codec. This is another type of Kolmogorov complexity estimate. While FLAC utilizes a lossless compression method, Ogg Vorbis uses an audio coding format which results in lossy compression. Ogg encoding was carried out using *ffmpeg* (https://ffmpeg.org/) with 16000 samples per second and 75 quality.
- **Event density:** The mean frequency (in hertz) of simultaneous harmonic, melodic and rhythmic events in the stimuli. As an example, a song with a fast rhythm would have a higher event density compared to a song with a slow rhythm. Event density was estimated with *MIRtoolbox* (https://www.jyu.fi/hytk/fi/laitokset/mutku/en/research/materials/mirtoolbox).

All measures were extracted from each 29-second long, 16000 Hz stimuli using a sliding window analysis with a window size of 8.82 s (12 TRs) and a hop size of 0.735 s (one TR). This resulted in 28 time points per stimulus per measure.

### Decoding analysis

In the decoding analysis, we used ridge regression to predict complexity measures from stimulus-evoked responses of voxels in ROIs. Let $x\in {\mathbb{R}}$ and ${\bf{y}}\in {{\mathbb{R}}}^{q}$ be a pair of a complexity measure and stimulus-evoked responses of *q* voxels in an ROI. We are interested in predicting *x* as a linear function of **y**:

3 $$x={{\boldsymbol{\beta }}}^{{\rm{{\rm T}}}}{\bf{y}}$$

where ***β*** is a vector of regression coefficients. Without loss of generality, we assume that *x* and **y** have zero mean and unit variance. We minimized the *L*^2^ penalized least squares loss function to estimate ***β***:

4 $${\boldsymbol{\beta }}=\mathop{{\rm{\arg }}\,{\rm{\min }}}\limits_{{\boldsymbol{\beta }}}\,[\frac{1}{n}\,\sum _{i=1}^{n}\,({x}^{(i)}-{{\boldsymbol{\beta }}}^{{\rm{{\rm T}}}}{{\bf{y}}}^{(i)})+\lambda \parallel {\boldsymbol{\beta }}{\parallel }_{2}^{2}]={({{\bf{Y}}}^{{\rm{{\rm T}}}}{\bf{Y}}+\lambda {{\bf{I}}}_{q})}^{-1}\,{{\bf{Y}}}^{{\rm{{\rm T}}}}{\bf{x}}$$

where *λ* ≥ 0 is a regularization coefficient and {**x**, **Y**} with **x** = (*x*^(1)^, …, *x*^(*n*)^)^Τ^ and **Y** = (**y**^(1)^, …, **y**^(*n*)^)^Τ^ is a training set consisting of *n x*-**y** pairs.

We used grid search to optimize *λ* as follows^59^: First, 100 linearly spaced values between 0.1 and min(*n*, *q*) − 0.1 were used to specify a set of effective degrees of freedom of the ridge regression fit. Then, Newton’s method was used to solve each value for *λ*. Finally, leave-one-out cross-validation on the training set was used to choose *λ*.

We estimated a separate decoding model for each complexity measure and all voxels in each ROI. We validated the decoding models on a held-out test set, which was at no point used for model estimation. We used the Pearson correlation coefficient between the ground truths and the predictions on the test set (*r*) as the validation metric.

### Encoding analysis

In the encoding analysis, we used linear regression to predict stimulus-evoked responses of voxels in ROIs from complexity measures. Let $x\in {\mathbb{R}}$ and $y\in {\mathbb{R}}$ be a pair of a complexity measure and a stimulus-evoked response of a voxel in an ROI. We are interested in predicting *y* as a linear function of *x*:

5 $$y={\beta }^{{\rm{{\rm T}}}}x$$

where *β* is a scalar regression coefficient. Without loss of generality, we assume that *x* and *y* have zero mean and unit variance. We minimized the least squares loss function to estimate *β*:

6 $$\beta =\mathop{{\rm{\arg }}\,{\rm{\min }}}\limits_{\beta }\,\frac{1}{n}\,\sum _{i=1}^{n}\,({y}^{(i)}-{\beta }^{{\rm{{\rm T}}}}{x}^{(i)})={({{\bf{x}}}^{{\rm{{\rm T}}}}{\bf{x}})}^{-1}{{\bf{x}}}^{{\rm{{\rm T}}}}{\bf{y}}$$

where {**x**, **y**} with **x** = (*x*^(1)^, …, *x*^(*n*)^)^Τ^ and **y** = (*y*^(1)^, …, *y*^(*n*)^)^Τ^ again denotes a training set.

We estimated a separate encoding model for each complexity measure and each voxel in each ROI. We validated the encoding models on a held-out test set, which was at no point used for model estimation. We used the Pearson correlation coefficient between the ground truths and the predictions on the test set (*r*) as the validation metric.

### Statistical analysis

We used permutation test to test the null hypothesis that *r* of a model is not different than the chance level as follows: First, the order of the ground-truths in the training and test sets were randomly shuffled. Then, a new model was estimated on the new training set and validated on the new test set. These steps were repeated 1000 times. The chance level was taken to be mean *r* of the new models. The *p*-value was taken to be the fraction of the new models whose *r* was greater than or equal to *r* of the old model. The null hypothesis was rejected if the *p*-value was less than or equal to 0.05.

### Hyperalignment

We performed the analyses on mean hyperaligned fMRI data^60^, which were obtained via the following iterative process: Before the first iteration, the training set fMRI data of the subject who has the largest number of voxels are assigned to a common representational space. At each iteration, i) the training set fMRI data of each subject are transformed to the common representational space with Procrustes transformation, ii) averaged along the subjects and iii) reassigned to the common representational space. After the final iteration, i) the training and test set fMRI data of each subject is transformed to the common representational space with Procrustes transformation and ii) averaged along the subjects. Note that the fMRI data from different experiments and/or ROIs were hyperaligned separately (Tables 3 and 4).

In other words, this process minimizes the Procrustes distance between fMRI data of different subjects with geometric transformations (rotation, translation and/or uniform scaling), which change the placement and the size but preserve the shape of the fMRI data of the different subjects. For example, consider the following toy three-trial fMRI data of two subjects who have two voxels each: {(1, 2), (3, 4), (5, 6)} and {(−24, 16) (−45, 35), (−66, 54)}. The former can be aligned to the latter with these transformations while keeping the shape of the former (line) intact as follows:

- Uniform scaling by 10: {(**1**, **2**), (**3**, **4**), (**5**, **6**)} → {(10, 20), (30, 40), (50, 60)}
- Translation by 5: {(10, 20), (30, 40), (50, 60)} → {(15, 25), (35, 45), (55, 65)}
- Rotation by *π*/2 radians: {(15, 25), (35, 45), (55, 65)} → {(−**25**, **15**) (−**45**, **35**), (−**65**, **55**)}

which results in a Procrustes distance (root sum square) of 2.

### Implementation details

Our fMRI preprocessing and analysis pipeline made use of standard analysis packages and custom scripts. Specifically, the following tools were used: Realignment and slice time correction were performed in SPM 12 (http://www.fil.ion.ucl.ac.uk/spm/) by using the default parameters for realignment, and scanning parameters and “Reference Slice” = 0 s for slice time correction. Detrending and standardization were performed in MATLAB. Note that the fMRI data from Experiment 1 are already provided in preprocessed format. We refer the reader to the original publication^1^ for more details on preprocessing of this data. Hyperalignment was performed in PyMVPA (http://www.pymvpa.org/) with default parameters. Encoding and decoding models as well as permutation tests were implemented with custom Python scripts (the relevant parameters are reported in the corresponding subsections). Projection of HCP MMP 1.0 parcellation to native space was done with Freesurfer (https://surfer.nmr.mgh.harvard.edu/) supplemented with custom scripts.

**Table 3:** Number of voxels in ROIs of the subjects from Experiment 1.

	V1	V2	V3	V4	LOC	FFA	PPA
S1	**1004**	1018	759	740	540	568	356
S2	757	944	810	544	834	435	316
S3	872	**1031**	861	754	**996**	928	496
S4	719	855	**929**	704	668	725	398
S5	659	891	907	**860**	566	**929**	**550**
**SH**	**1004**	**1031**	**929**	**860**	**996**	**929**	**550**

SH denotes mean hyperaligned fMRI data.

**Table 4:** Number of voxels in ROIs of the subjects from Experiment 2.

	A1	LBelt	MBelt	PBelt	RI	A4	A5	STSdp	STSda	STSvp	STSva	STGa	TA2
S1	40	71	73	119	87	241	269	177	**288**	201	191	183	117
S2	39	67	71	87	92	162	172	131	142	153	138	138	112
S3	**81**	90	**128**	163	**111**	320	285	**206**	197	**274**	**200**	154	**165**
S4	70	**103**	109	**173**	102	**322**	**288**	197	220	227	199	143	147
S5	54	95	89	142	93	244	254	151	198	189	180	146	126
S6	33	59	87	104	71	221	173	144	131	187	118	132	128
S7	51	91	88	129	82	258	249	139	207	251	164	**183**	99
S8	62	83	98	132	89	254	281	163	205	181	156	139	140
**SH**	**81**	**103**	**128**	**173**	**111**	**322**	**288**	**206**	**288**	**274**	**200**	**183**	**165**

SH denotes mean hyperaligned fMRI data.

### Data availability

The dataset analysed during the current study (Experiment 1) is available in the ATR brainliner repository, http://brainliner.jp/data/brainliner/Generic_Object_Decoding. The dataset generated and analysed during the current study (Experiment 2) is available from the corresponding author on reasonable request.

## Results

### Experiment 1

We first examined the relationship between the measures of visual complexity by calculating bivariate Pearson correlation coefficients between each measure (Table 5). All three measures were significantly correlated with each other (*p* < 0.05). Highest correlation was between gradient and PNG (*r* = 0.86), whereas the lowest correlation was observed between gradient and self-similarity (*r* = 0.48).

**Table 5:** Experiment 1 - Bivariate correlations (*r*) between visual complexity measures.

	Gradient	PNG	Self-similarity
Gradient	1.00	0.86	0.48
PNG	0.86	1.00	0.65
Self-similarity	0.48	0.65	1.00

**Figure 1:**
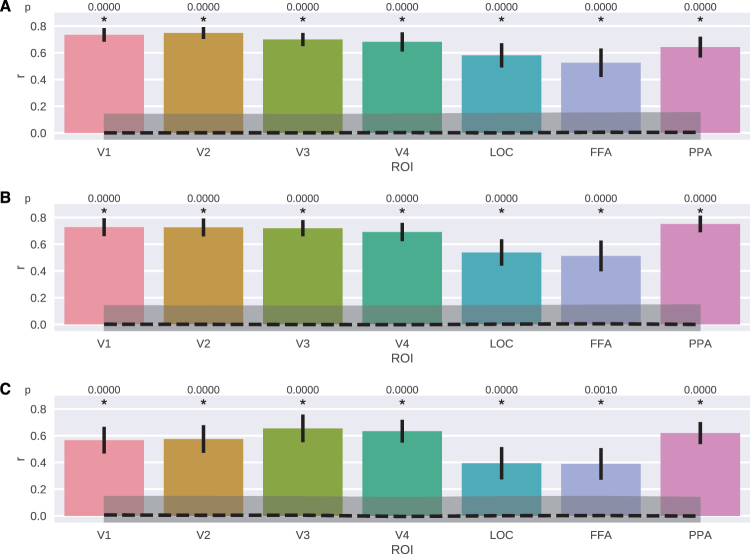
Experiment 1 - Results of decoding visual complexity measures from the ROIs in the visual cortex. (**A**) Gradient. (**B**) PNG. (**C**) Self-similarity. Bars and error bars show decoding performance and ±1 SEM, respectively. Dashed line and shaded region show chance level and ±1 SEM around *r* = 0, respectively. SEM is computed with bootstrapping (1000 iterations).

Next, we performed the decoding analysis on the hyperaligned data of the five subjects. For each visual complexity measure, we predicted the value of the complexity measure from the stimulus-evoked responses of voxels in the ROIs from the visual cortex (Table 2) using ridge regression. Note that this is a multivariate analysis, such that in order to predict the values of a complexity measure of stimuli, all voxel responses in a ROI are used at the same time. Figure 1 shows the visual complexity decoding results. Remarkably, from all ROIs in the visual cortex, it was possible to predict all three visual complexity measures significantly above chance level (p $\ll$ 0.001) with a maximum correlation between the predicted values and actual complexity measure values of *r* = 0.75 for PNG measure in PPA and a minimum correlation of *r* = 0.40 for self-similarity measure in FFA. Among all three complexity measures, PNG had the highest (*r* = 0.67) and self-similarity had the lowest (*r* = 0.56) average correlation over the ROIs. For all three measures, LOC and FFA regions had the lowest decoding performance, and among the ROIs in the higher visual cortex, all complexity measures could be predicted with highest accuracy from PPA (*r* = 0.65, 0.75, 0.62 for gradient, PNG and self-similarity measures, respectively).

**Figure 2:**
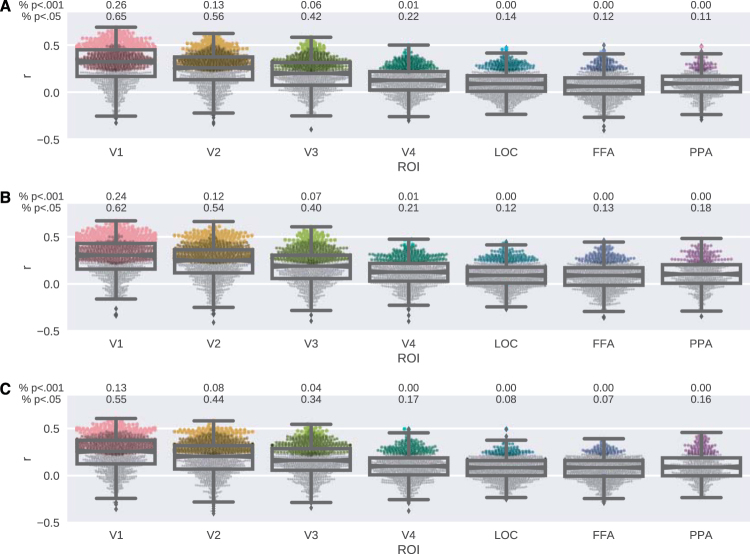
Experiment 1 - Distributions of encoding performance over individual voxels in visual ROIs. (**A**) Gradient. (**B**) PNG. (**C**) Self-similarity. Boxes show interquartile range. Notches show second quartile. Whiskers show ±1.5 interquartile range. Points show encoding performance of individual voxels. Colors show *p*-values (black: outlier; gray: *p* ≥ 0.05; dark: *p* < 0.05; light: *p* < 0.001).

Then, we evaluated how well the stimulus-evoked responses in ROIs could be predicted from the visual complexity measures using linear regression (Fig. 2). Note that unlike the decoding analyses which were multivariate, encoding analyses are univariate, such that we estimate a separate encoding model for each complexity measure and each voxel in each ROI. For all complexity measures, we observed that the encoding performance decreased along the visual hierarchy such that the percentage of voxels whose responses were predicted significantly above chance (*p* < 0.05) was highest in V1 (65%, 62% and 55% for gradient, PNG and self-similarity measures, respectively) and the lowest in PPA (11% for the gradient measure), LOC (12% for the PNG measure) or FFA (7% for the self-similarity measure). Furthermore, for all of the investigated visual complexity measures, the encoding performance was higher in the ROIs in the lower visual cortex and relatively low in the ROIs in the higher visual cortex and V4. Compared to the gradient measure, the stimulus-evoked voxel responses in the PPA region could be better predicted from the PNG and self-similarity measures.

**Figure 3:**
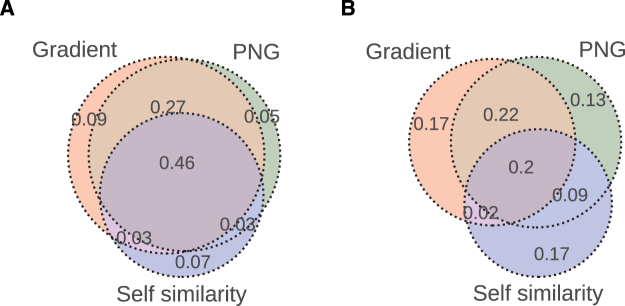
Experiment 1 - Overlap between different visual complexity measures in the visual cortex. (**A**) Ratio of overlapping voxels whose responses were significantly predicted in the lower visual cortex. (**B**) Ratio of overlapping voxels whose responses were significantly predicted in the higher visual cortex and V4.

Next, we investigated the overlap between different visual complexity measures in terms of the number of voxels whose responses were significantly predicted in the lower and higher visual cortices (Fig. 3). In the lower visual cortex, the amount of overlap between the significantly predicted voxel responses of all three visual complexity measures was relatively high with 46% of all significant voxels. This overlap reduced to 20% in ROIs in the higher visual cortex and V4. Furthermore, the amount of overlap between the gradient and PNG measures was very high (73%) in the lower visual cortex. This overlap reduced to 44% in the ROIs in the higher visual cortex. Out of all the significantly predicted voxels in the higher visual cortex, 17% were only sensitive to the gradient measure, whereas this number was 13% for the PNG measure and 17% for the self-similarity measure.

**Figure 4:**
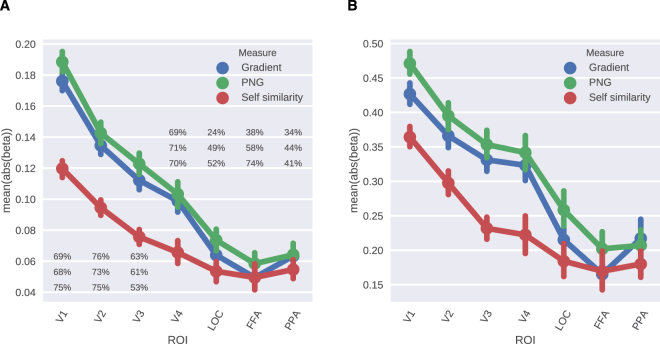
Experiment 1 - (**A**) Mean absolute beta coefficients over the significant voxels in the visual ROIs. Percentages show the percentage of positive beta coefficients corresponding to each complexity measure: Gradient, PNG and Self-similarity, from top to bottom, respectively. (**B**) Mean absolute normalized beta (beta/ r) over the significant voxels in the visual ROIs. Error bars show ±1 SEM.

Finally, we investigated the sensitivity of the voxels whose responses were significantly predicted (*p* < 0.05) in each ROI to the measures of visual complexity by calculating the mean absolute beta coefficient (i.e. slope) of the regression models for each ROI. The beta coefficients show the extent to which the response of a voxel is modulated per one standard deviation change in the complexity value of the stimulus. Figure 4 shows the results of these analyses. We observed that while in V1 the mean slope was the highest, it decreased almost gradually as we moved to higher visual areas, suggesting that the representations of complexity became coarser along the visual hierarchy. Since this pattern was very similar to the encoding performance, we further calculated the slopes controlled for the encoding performance by dividing the beta coefficients by the corresponding correlation coefficients (Panel B in Fig. 4). This did not change the observed pattern, confirming that the slopes of betas were indeed indicative of the sensitivity of complexity representations of voxels rather than just reflecting the encoding performance. Another interesting result that we found was that large portions of the voxels in all ROIs of the visual cortex had negative slopes, such that their response increased as the complexity of the image decreased. While a majority of the voxels in the lower visual areas and V4 had positive slopes, in the higher visual areas voxels selective to simplicity rather than complexity were more common.

### Experiment 2

First, to understand the relationship between the auditory complexity measures, we calculated bivariate Pearson correlation coefficients between each measure (Table 6). All three measures were significantly correlated with each other (*p* < 0.05). Highest correlation was between the two compression measures FLAC and Ogg (*r* = 0.66), whereas the lowest correlation was observed between event density and Ogg (*r* = 0.23).

**Table 6:** Experiment 2 - Bivariate correlations (*r*) between auditory complexity measures.

	Event density	FLAC	Ogg
Event density	1.00	0.45	0.23
FLAC	0.45	1.00	0.66
Ogg	0.23	0.66	1.00

**Figure 5:**
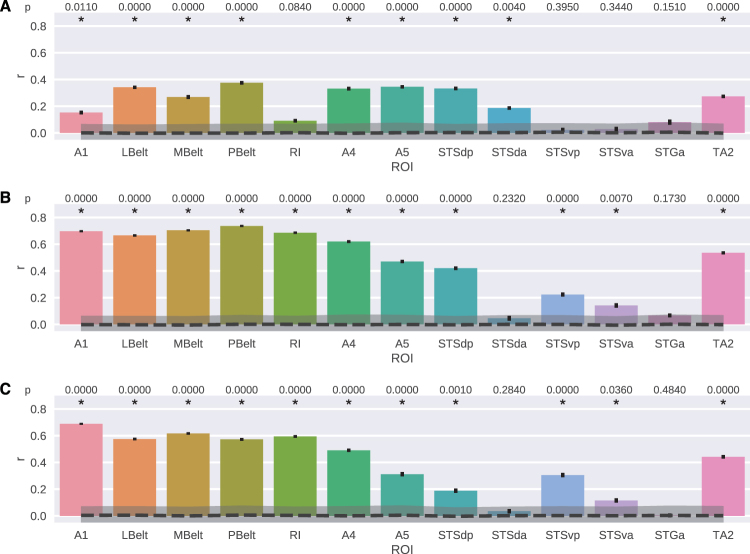
Experiment 2 - Results of decoding auditory complexity measures from the ROIs in the auditory cortex. (**A**) Event density. (**B**) FLAC. (**C**) Ogg. Bars and error bars show decoding performance and ±1 SEM, respectively. Dashed line and shaded region show chance level and ±1 SEM around *r* = 0, respectively. SEM is computed with bootstrapping (1000 iterations).

Next, we performed the decoding analysis on the hyperaligned data of the eight subjects. That is, for each one of the auditory complexity measures, we predicted the value of the complexity measure from the stimulus-evoked responses of voxels in the ROIs from the early auditory and auditory association cortices (Table 2) using ridge regression. The results of these analyses are presented in Fig. 5. The most striking (but not unexpected) result was that for the two file compression measures FLAC and Ogg, the general pattern of the decoding performances in ROIs were very similar with each other, but not very similar to the event density measure. For the event density measure, decoding performance was worse than those of the compression measures in all ROIs except for the STSda region. Overall, the highest decoding performance was obtained for the FLAC measure with the highest correlation results in the PBelt region (*r* = 0.74, *p* $\ll$ 0.001) followed by MBelt (*r* = 0.70, *p* $\ll$ 0.001) and A1 (*r* = 0.70, *p* $\ll$ 0.001) regions. For the two compression measures, all ROIs in the early auditory cortices were decoded significantly above chance, with all *r* values above 0.57. In the early auditory cortex, largest differences between the decoding performance were observed in the RI region between the event density and both of the compression measures, where for event density the predictions were not better than chance and for FLAC and Ogg, correlations were rather high (*r* = 0.69, *p* $\ll$ 0.001 and *r* = 0.59, *p* $\ll$ 0.001 for FLAC and Ogg, respectively). Conversely, in STSda, neither FLAC nor Ogg measure could be predicted significantly above the chance level, whereas event density was decoded significantly (*r* = 0.19, *p* < 0.05). Among the ROIs in the auditory association cortex, the compression measures could be best predicted from the voxels in A4 (*r* = 0.62, *p* $\ll$ 0.001 and *r* = 0.49, *p* $\ll$ 0.001 for FLAC and Ogg, respectively) and TA2 (*r* = 0.53, *p* $\ll$ 0.001 and *r* = 0.44, *p* $\ll$ 0.001 for FLAC and Ogg, respectively) regions. The only ROI that none of the auditory complexity measures could be predicted from was STGa.

**Figure 6:**
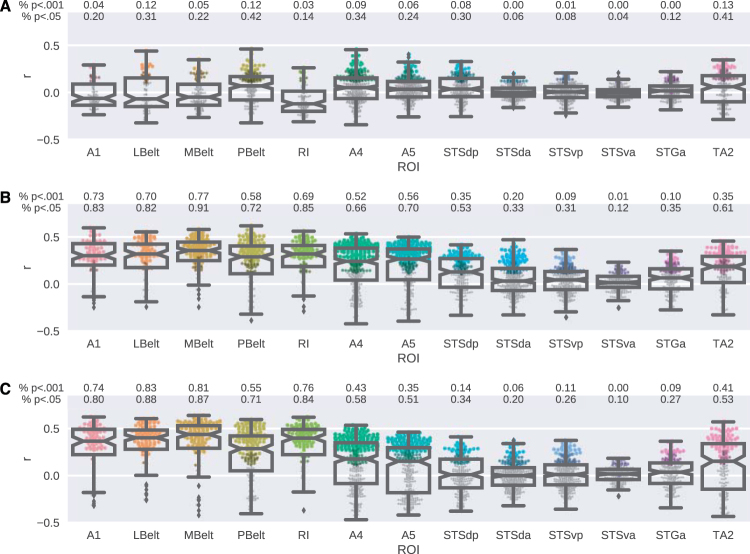
Experiment 2 - Distributions of encoding performance over individual voxels in auditory ROIs. (**A**) Event density. (**B**) FLAC. (**C**) Ogg. Boxes show interquartile range. Notches show second quartile. Whiskers show ±1.5 interquartile range. Points show encoding performance of individual voxels. Colors show *p*-values (black: outlier; gray: *p* ≥ 0.05; dark: *p* < 0.05; light: *p* < 0.001).

Following the decoding analyses, we performed encoding analysis, again on the hyperaligned data of the eight subjects, to evaluate how well the stimulus-evoked responses in ROIs could be predicted from the auditory complexity measures using linear regression. The results of the encoding analyses are presented in Fig. 6. Similar to the decoding results, the general pattern of the encoding performances of the two compression measures were very high and resembled each other. A large majority of the voxel responses in the early auditory cortices could be predicted significantly above chance level (*p* < 0.05) using FLAC and Ogg measures. The percentage of the significantly predicted voxel responses ranged from 72% to 91% for the FLAC measure and from 71% to 87% for the Ogg measure. Among the ROIs in the auditory associative cortex best encoding performance was in A5 for FLAC and A4 for Ogg, whereas the worst performance was in STSva for both compression measures. The encoding performance of the regression models using the event density measure was relatively low in all ROIs with the highest percentage of significantly above chance level (*p* < 0.05) predictions in PBelt (43%), A4 (34%) and TA2 (41%) regions.

**Figure 7:**
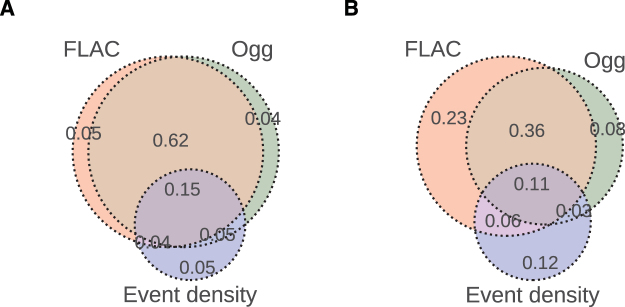
Experiment 2 - Overlap between different auditory complexity measures in the auditory cortex. (**A**) Ratio of overlapping voxels whose responses were significantly predicted in the early auditory cortex. (**B**) Ratio of overlapping voxels whose responses were significantly predicted in the auditory association cortex.

Next, we investigated the overlap between different auditory complexity measures in terms of the number of voxels whose responses were significantly predicted in the early auditory and auditory association cortices (Fig. 7). The amount of overlap between the significantly predicted voxel responses of FLAC and Ogg measures was very high (77%) in the early auditory cortex. This overlap reduced to 47% in the ROIs in the auditory associative cortex. Out of all the significantly predicted voxels in the auditory associative cortex, 29% were only sensitive to the FLAC measure, whereas this number was 10% for the Ogg measure and 12% for the event density. The voxels that were significantly predicted by all three measures made up 15% of all significant voxels in the early auditory cortex and 11% in the auditory associative cortex.

**Figure 8:**
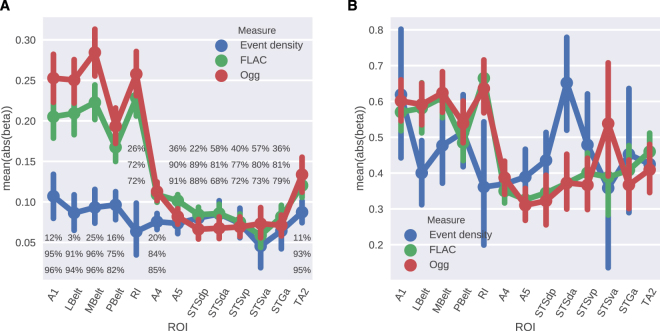
Experiment 2 - (**A**) Mean absolute beta coefficients over the significant voxels in the auditory ROIs. Percentages show the percentage of positive beta coefficients corresponding to each complexity measure: Event density, FLAC and Ogg, from top to bottom, respectively. (**B**) Mean absolute normalized beta (beta/ r) over the significant voxels in the auditory ROIs. Error bars show ±1 SEM.

Finally, we looked at how the mean beta coefficients varied between different ROIs (Fig. 8). We observed that there was a large difference between the early auditory cortex and the auditory association cortex for the FLAC and Ogg measures, such that the early regions had a finer sensitivity to complexity and the associative regions had a coarser sensitivity to changes in complexity levels. For the event density measure, the mean beta levels were low in all ROIs. When controlled for the encoding performance (Panel B in Fig. 8) the differences between the early and associative auditory cortices remained similar except for in the STSva region for the Ogg measure which had a large variability. This analysis also revealed that the STSda region showed high sensitivity to the event density measure. Overall, the mean betas of the event density measure showed relatively larger variability both between and within ROIs. Regarding the direction of the relationship between complexity and voxel responses, for the compression complexity measures FLAC and Ogg (unlike in the visual cortex) the majority of voxels in all auditory ROIs had positive beta coefficients, i.e they responded more as the stimulus complexity increased. However for the event density measure, most voxels had negative slopes.

## Discussion

In this study, we investigated the neural representations of image and music complexity in the human visual and auditory cortices. To this end, we performed univariate encoding and multivariate decoding analyses of fMRI data from two different experiments measuring the stimulus-evoked BOLD responses to large collections of photograph and music stimuli. In Experiment 1, we found that visual complexity was represented throughout the visual cortex with decreasing sensitivity from lower to higher visual areas. While all regions in the lower visual cortex were highly responsive to stimulus complexity, in the higher visual cortex, PPA was the most responsive region to visual complexity of photographs. Voxels representing complexity in the lower visual cortex mostly showed increased activity with increased complexity, whereas approximately half of those in the higher visual areas showed decreased activity as image complexity increased. In the case of representations of auditory complexity, in Experiment 2, we found that encoding and decoding performances and sensitivity of voxels were high for the early auditory cortex and on average lower for the regions in the auditory association cortex. Among the ROIs in the auditory association cortex, A4 and TA2 were the most responsive regions to stimulus complexity. Furthermore, we determined that representations of event density in music were less pronounced compared to those of Kolmogorov complexity measures throughout the auditory cortex.

The differences between the neural representations of different complexity measures, which are also suggested by varying degrees of correlations between these measures, became more pronounced after a fine-grained analysis of the results. In both visual and auditory cortices, we showed that in the earlier sensory areas, the overlap between voxels representing different complexity measures were higher than the overlap in the areas higher in the sensory hierarchy. This result implies that voxels coding for different complexity dimensions become more specialized along the visual and auditory hierarchy.

Furthermore, both in the auditory and the visual cortices, the sensitivity of voxels to changes in complexity – measured by the magnitudes of the regression slopes of the voxel encoding models – decreased along the sensory hierarchy. While this decrease was gradual in the visual cortex, it was more abrupt starting at the A4 region in the auditory association cortex, and in the auditory cortex was only observed for the Kolmogorov complexity measures. These results are reminiscent of the representational gradients of other sensory stimulus features such as semantic features of movies^61^ and speech^62^, and task-optimized features of images^63^, movies^64^ or music^65^, which further suggest a functional organization in terms of gradients rather than patches.

Another difference between the complexity representations in the two sensory cortices was observed between the number of voxels showing increased activity with increased complexity. In the auditory cortex, the responses of voxels were mostly positively correlated with complexity, whereas in the higher visual cortex, around half of the voxels had negative beta coefficients indicating increased activity in response to increased stimulus simplicity rather than complexity.

We found that from PPA, all of the tested complexity measures could be decoded with a very high accuracy but this was not the case for the encoding analysis. We believe that this suggests the presence of distributed representations of complexity in PPA (cf. Park *et al*.^66^) rather than single voxels encoding for information regarding scene complexity. Possibly, our multivariate decoding approach allowed us to make accurate predictions about the complexity of the stimulus, whereas our univariate encoding analysis did not allow to capture the distributed representations of complexity.

The decoding accuracy in PPA was on par with the regions in the early visual cortex and was much better than other higher level regions such as FFA and LOC. This result might seem surprising at first glance, however it is actually not unexpected given that PPA is primarily responsible for representing scenes^67,68^, for which complexity is one of the defining properties that allows identifying different scene categories^69^. On the other hand, such a relationship has not been established to the same extent for objects and faces. Our results are in line with the accepted functional role of the PPA in scene processing and support previous behavioural results showing that scene identification utilizes global image properties^70,71^.

The auditory association regions that we identified to represent music complexity largely overlapped with the regions that have been shown to activate during story listening and auditory math tasks, whereas we observed no strong correspondences with the story - math contrast^58^. As seen in Fig. 5 decoding of complexity levels from anterior STS and STG regions were rather unsuccessful, i.e. these regions performed either not or merely significantly different from chance. For example, in STSda, only event density could be significantly decoded and this was with a low amount of correlation. Similarly in STSva only FLAC and Ogg could be decoded with a performance that was merely significantly above chance and no measure could be predicted from STGa. Moving from anterior to posterior regions, the decoding performance of the ROIs increased, such that performance in both STSvp and STSdp was significant, and in A4 and A5 regions, which lie on posterior STG, it was the highest among the areas in the auditory association cortex. (Left) posterior STG and STS regions have been shown to process syntactic complexity of language in several previous studies (for a review, see^72^). In most of these studies syntactic complexity is investigated by comparing list of words to sentences, sentences with simple syntactic structures to those with complex ones^72^. Our results show that posterior STG and STS regions not only process syntactic complexity, but also they represent music complexity.

In the auditory association cortex, besides the ROIs on posterior STG and STS, we identified TA2 (which lies on planum polare) as a region that represents complexity well. In terms of speech and other complex sounds, the function of this region has not been well established, however it is known to show greater activity in response to music, compared to vocal and speech sounds^47^. Our results demonstrate that TA2 is a region which also represents complexity of music.

Event density has been suggested as a good measure of the complexity of songs^19,73^. However, based on our results, event density showed different neural representations than the Kolmogorov complexity measures in the auditory cortex. Furthermore, event density was less well encoded and decoded by our approaches compared to the FLAC and Ogg measures.

We have used three different complexity measures per sensory modality so as to not overlook the multifaceted nature of complexity as discussed in the Introduction section. Both in the visual and auditory domains, this multifaceted nature of complexity makes it difficult to perfectly define complexity and select a “best” measure, especially when estimating the complexity levels of naturalistic stimuli. By reporting the results of our analysis for a selection of computational complexity measures, we aimed to provide a greater insight into how different measures of complexity are processed in the human brain. We hope that the differences among the different measures that we report here will be a useful resource for future studies.

In this study, our goal was to establish a direct, predictive relationship between objective stimulus complexity and stimulus-evoked brain activations at single subject level rather than making inferences at population level in accordance with previous neural encoding and decoding studies in the literature^49,63,74,75^. As such, we used data sets with a very large number of data points per subject (approximately 3 h and 9 h per subject) but a small number of subjects in total (eight and five subjects in total) for training and testing predictive models on separate datasets. Therefore, we performed statistical analyses and tests at single subject level. As a result, we were able to establish such a direct, predictive relationship. However, it should be noted that making inferences at population level would require a larger group study.

Literature in art perception suggests a strong link between complexity and aesthetic responses. A previous study investigating aesthetic responses to mathematical formulae indirectly provides some insight into how elegance (or simplicity) of highly intellectual and abstract concepts are processed in the brain^76^. The authors of the study found that the beautiful mathematical formulae (which in many case were the formulae which were simple yet meaningful) activated the A1 region of the medial orbito-frontal cortex, which is known also to activate in response to beauty of art. However, this study investigated conceptual or mathematical elegance rather than perceptual simplicity. Therefore, it would still be interesting to investigate how stimulus complexity manifests itself throughout the brain (besides the currently investigated sensory cortices) in response to artworks, images and music of varying complexity levels. Moreover, an interesting next step would be to investigate the effects of additional factors such as scene preferences^77^ on the neural representations of stimulus complexity. Finally, while all of the objective computational complexity measures such as Kolmogorov complexity (PNG, FLAC and Ogg), gradient, self-similarity and event density that were employed in this study are established estimates of subjective complexity of auditory and visual stimuli, subjectively rated stimulus complexity can be investigated in future studies.

## Electronic supplementary material

Supplementary Information
