# Supplementary material for: Representations of naturalistic stimulus complexity in early and associative visual and auditory cortices
Source: Sci Rep. 2018 Feb 21;8:3439. doi: 10.1038/s41598-018-21636-y (PMC5821852; doi:10.1038/s41598-018-21636-y)
Supplement: Supplementary file 1 — Supplementary Information [file 41598_2018_21636_MOESM1_ESM.pdf]

Representations of naturalistic stimulus complexity in early and associative visual and auditory cortices

Yağmur Güçlütürk[1,*], Umut Güçlü[1], Marcel van Gerven[1], and Rob van Lier[1]

[1]Radboud University, Donders Institute for Brain, Cognition and Behaviour, Nijmegen, the Netherlands
[*]y.gucluturk@donders.ru.nl

Supplementary Information

Image Categories

Category 1: French horn, horn. Category 2: Frisbee. Category 3: Kalashnikov. Category 4: Segway, Segway Human Transporter, Segway HT. Category 5: airliner. Category 6: airship, dirigible. Category 7: baby buggy, baby carriage, carriage, perambulator, pram, stroller, go-cart, pushchair, pusher. Category 8: backpack, back pack, knapsack, packsack, rucksack, haversack. Category 9: barrow, garden cart, lawn cart, wheelbarrow. Category 10: baseball bat, lumber. Category 11: baseball glove, glove, baseball mitt, mitt. Category 12: basket, basketball hoop, hoop. Category 13: bat, chiropteran. Category 14: bathtub, bathing tub, bath, tub. Category 15: beacon, lighthouse, beacon light, pharos. Category 16: bear. Category 17: beer mug, stein. Category 18: binoculars, field glasses, opera glasses. Category 19: birdbath. Category 20: bonsai. Category 21: bowling ball, bowl. Category 22: bowling pin, pin. Category 23: boxing glove, glove. Category 24: bulldozer, dozer. Category 25: butterfly. Category 26: camel. Category 27: camera tripod. Category 28: cannon. Category 29: canoe. Category 30: car wheel. Category 31: centipede. Category 32: cereal box. Category 33: chimpanzee, chimp, Pan troglodytes. Category 34: clasp knife, jackknife. Category 35: cockroach, roach. Category 36: coffee mug. Category 37: coffin, casket. Category 38: common iguana, iguana, Iguana iguana. Category 39: common raccoon, common racoon, coon, ringtail, Procyon lotor. Category 40: compact disk, compact disc, CD. Category 41: computer keyboard, keypad. Category 42: computer monitor. Category 43: conch. Category 44: cormorant, Phalacrocorax carbo. Category 45: covered wagon, Conestoga wagon, Conestoga, prairie wagon, prairie schooner. Category 46: cowboy hat,

ten-gallon hat. Category 47: crab. Category 48: dial telephone, dial phone. Category 49: diskette, floppy, floppy disk. Category 50: dog, domestic dog, Canis familiaris. Category 51: dolphin. Category 52: domestic llama, Lama peruana. Category 53: dress hat, high hat, opera hat, silk hat, stovepipe, top hat, topper, beaver. Category 54: duck. Category 55: dumbbell. Category 56: earphone, earpiece, headphone, phone. Category 57: electric guitar. Category 58: elephant. Category 59: elk, European elk, moose, Alces alces. Category 60: fighter, fighter aircraft, attack aircraft. Category 61: fire engine, fire truck. Category 62: fire extinguisher, extinguisher, asphyxiator. Category 63: flashlight, torch. Category 64: football helmet. Category 65: frog, toad, toad frog, anuran, batrachian, salientian. Category 66: frying pan, frypan, skillet. Category 67: gas pump, gasoline pump, petrol pump, island dispenser. Category 68: ghetto blaster, boom box. Category 69: giraffe, camelopard, Giraffa camelopardalis. Category 70: goat, caprine animal. Category 71: goldfish, Carassius auratus. Category 72: golf ball. Category 73: goose. Category 74: gorilla, Gorilla gorilla. Category 75: grand piano, grand. Category 76: grape. Category 77: grasshopper, hopper. Category 78: gravestone, headstone, tombstone. Category 79: greyhound. Category 80: guitar pick. Category 81: gym shoe, sneaker, tennis shoe. Category 82: hammock, sack. Category 83: hand calculator, pocket calculator. Category 84: harmonica, mouth organ, harp, mouth harp. Category 85: harp. Category 86: harpsichord, cembalo. Category 87: hawksbill turtle, hawksbill, hawkbill, tortoiseshell turtle, Eretmochelys imbricata. Category 88: helicopter, chopper, whirlybird, eggbeater. Category 89: homo, man, human being, human. Category 90: horse, Equus caballus. Category 91: horseshoe crab, king crab, Limulus polyphemus, Xiphosurus polyphemus. Category 92: hot tub. Category 93: hot-air balloon. Category 94: hourglass. Category 95: housefly, house fly, Musca domestica. Category 96: hummingbird. Category 97: iPod. Category 98: ibis. Category 99: joystick. Category 100: kangaroo. Category 101: kayak. Category 102: ketch. Category 103: killer whale, killer, orca, grampus, sea wolf, Orcinus orca. Category 104: knife. Category 105: knob, boss. Category 106: laptop, laptop computer. Category 107: lathe. Category 108: lawn mower, mower. Category 109: leopard, Panthera pardus. Category 110: light bulb, lightbulb, bulb, incandescent lamp, electric light, electric-light bulb. Category 111: mailbox, letter box. Category 112: mandolin. Category 113: marimba, xylophone. Category 114: megaphone. Category 115: menorah. Category 116: microscope. Category 117: microwave, microwave oven. Category 118: minaret. Category 119: miniature fan palm, bamboo palm, fern rhapis, Rhapis excelsa. Category 120: motorcycle, bike. Category 121: mountain bike, all-terrain bike, off-roader. Category 122: mouse, computer mouse. Category 123: mushroom. Category 124: mussel. Category 125: necktie, tie. Category 126: obelisk. Category 127: octopus, devilfish. Category 128: ostrich, Struthio camelus. Category 129: owl, bird of Minerva, bird of night, hooter. Category 130: palm, palm tree. Category 131: paper clip, paperclip, gem clip. Category 132: penguin. Category 133: personal digital assistant, PDA, personal organizer, personal organiser, organizer, organiser. Category 134: photocopier. Category 135: pincer, pair of pincers, tweezer, pair of tweezers. Category 136:

pitcher, ewer. Category 137: planchet, coin blank. Category 138: pool table, billiard table, snooker table. Category 139: porcupine, hedgehog. Category 140: praying mantis, praying mantid, Mantis religioso. Category 141: projector. Category 142: radio telescope, radio reflector. Category 143: refrigerator, icebox. Category 144: revolver, six-gun, six-shooter. Category 145: rifle. Category 146: roulette wheel, wheel. Category 147: saddle. Category 148: school bus. Category 149: scorpion. Category 150: screwdriver. Category 151: sextant. Category 152: shirt. Category 153: shredder. Category 154: skateboard. Category 155: skunk, polecat, wood pussy. Category 156: skyscraper. Category 157: snail. Category 158: snake, serpent, ophidian. Category 159: snowmobile. Category 160: soccer ball. Category 161: sock. Category 162: soda can. Category 163: spectacles, specs, eyeglasses, glasses. Category 164: speedboat. Category 165: spider. Category 166: spoon. Category 167: stained-glass window. Category 168: starfish, sea star. Category 169: steering wheel, wheel. Category 170: stirrup, stirrup iron. Category 171: sunflower, helianthus. Category 172: swan. Category 173: sword, blade, brand, steel. Category 174: syringe. Category 175: tambourine. Category 176: teapot. Category 177: telephone booth, phone booth, call box, telephone box, telephone kiosk. Category 178: tennis ball. Category 179: tennis racket, tennis racquet. Category 180: tepee, tipi, teepee. Category 181: theodolite, transit. Category 182: toaster. Category 183: toaster oven. Category 184: tomato. Category 185: treadmill. Category 186: triceratops. Category 187: tricycle, trike, velocipede. Category 188: trilobite. Category 189: true toad. Category 190: tuning fork. Category 191: umbrella. Category 192: videocassette recorder, VCR. Category 193: washer, automatic washer, washing machine. Category 194: watch, ticker. Category 195: watermelon. Category 196: welder's mask. Category 197: windmill. Category 198: wine bottle. Category 199: yarmulke, yarmulka, yarmelke. Category 200: zebra.

Music Tags

Tag 1: airy. Tag 2: ambient. Tag 3: arabic. Tag 4: beats. Tag 5: bells. Tag 6: blues. Tag 7: cello. Tag 8: classical. Tag 9: country. Tag 10: dance. Tag 11: dark. Tag 12: drums. Tag 13: eastern. Tag 14: eerie. Tag 15: electric. Tag 16: electric guitar. Tag 17: electro. Tag 18: fast. Tag 19: female singing. Tag 20: flutes. Tag 21: guitars. Tag 22: hard rock. Tag 23: harp. Tag 24: harpsichord. Tag 25: heavy metal. Tag 26: horns. Tag 27: indian. Tag 28: instrumental. Tag 29: jazz. Tag 30: jazzy. Tag 31: loud. Tag 32: man singing. Tag 33: metal. Tag 34: middle eastern. Tag 35: new age. Tag 36: no guitars. Tag 37: no singing. Tag 38: noise. Tag 39: opera. Tag 40: oriental. Tag 41: piano. Tag 42: pop. Tag 43: quiet. Tag 44: rock. Tag 45: sax. Tag 46: singing. Tag 47: sitar. Tag 48: slow. Tag 49: soft. Tag 50: soft rock. Tag 51: strange. Tag 52: strings. Tag 53: synth. Tag 54: techno. Tag 55: trumpet. Tag 56: violins. Tag 57: weird. Tag 58: wind. Tag 59: world.
